# Serum Osmolality as a Predictor of Renal Function Decline: A Retrospective Cohort Study

**DOI:** 10.3390/jcm13216505

**Published:** 2024-10-30

**Authors:** Jheng-Jia Wu, Chun-Wu Tung, Chun-Wei Lin, Jui-Chu Huang, Jen-Tsung Yang, Yuan-Hsiung Tsai, Yun-Shing Peng

**Affiliations:** 1 Division of Endocrinology and Metabolism, Department of Internal Medicine, Chang Gung Memorial Hospital, Chiayi 61363, Taiwan; ssjump@cgmh.org.tw (J.-J.W.); ytchao@cgmh.org.tw (J.-C.H.); 2 College of Medicine, Chang Gung University, Taoyuan 33302, Taiwan; yljwty@cgmh.org.tw; 3 Department of Nephrology, Chang Gung Memorial Hospital, Chiayi 61363, Taiwan; p122219@cgmh.org.tw; 4 Chang Gung Medical Education Research Centre, Taoyuan 33302, Taiwan; 5 Department of Biochemical Science and Technology, National Chiayi University, Chiayi 61363, Taiwan; 6 Department of Diagnostic Radiology, Chang Gung Memorial Hospital, Chiayi 61363, Taiwan; f3700069@cgmh.org.tw; 7 Division of Endocrinology and Metabolism, Department of Internal Medicine, Chang Gung Memorial Hospital, Yunlin 638, Taiwan; 8 Graduate Institute of Clinical Medical Sciences, College of Medicine, Chang Gung University, Chiayi 613, Taiwan; 9 Department of Neurosurgery, Chang Gung Memorial Hospital, Chiayi 61363, Taiwan

**Keywords:** osmolality, dehydration, chronic kidney disease, renal function decline, general population

## Abstract

**Background and Aims:** Dehydration is a prevalent and costly healthcare concern, linked to heightened risks of acute kidney injury and in-hospital mortality. Despite its significance, limited evidence exists regarding its prevalence and correlation with renal function decline in apparently healthy individuals. This retrospective cohort study aimed to investigate the prevalence and association of dehydration with renal function decline and the development or progression of chronic kidney disease (CKD) in the general population. **Methods:** The medical records of subjects undergoing annual health check-ups from 2016 to 2019 at a single center in Taiwan were analyzed, and those with CKD stage V, insufficient data, or an increased estimated glomerular filtration rate (eGFR) were excluded. Serum osmolality, eGFR, and relevant parameters were measured. Logistic regression and Kaplan–Meier analyses were used to assess associations between osmolality and CKD-related outcomes. **Results:** Among the 4449 eligible subjects, those in the higher osmolality quartiles had an elevated risk of CKD or CKD progression. Multivariate analyses identified age, systolic blood pressure, serum osmolality, uric acid, proteinuria, and a history of diabetes as independent risk factors, with high-density lipoprotein being protective. Cumulative incidence curves demonstrated a significant increase in the risk of CKD with increasing osmolality levels. Restricted cubic spline analyses confirmed a nonlinear relationship between osmolality and CKD risk. **Conclusions:** Elevated serum osmolality independently predicted renal function decline and CKD development in apparently healthy individuals, and this effect persisted after adjusting for established risk factors. Our findings underscore the importance of addressing dehydration as a modifiable risk factor for CKD.

## 1. Introduction

Dehydration describes a deficiency in total body water, and it is both a prevalent and costly issue in hospitals and long-term care institutes [1,2]. The term “dehydration” is derived from the Greek word “hudōr”, meaning ‘water’. According to the World Health Organization (WHO), dehydration results from an excessive loss of water from the body. Classically, the medical literature distinguishes between two forms of acute fluid loss: dehydration, which primarily affects intracellular compartments, and volume depletion, which refers to the loss of extracellular fluid and the interstitial compartments [3,4]. More expansive definitions are offered on the basis of differing physiological effects on the extracellular compartment: hypotonic, isotonic, or hypertonic dehydration [5].

Dehydration can manifest as either a short-term effect or a long-term condition, which is often mild but may lead to adverse health outcomes if not addressed [6]. Dehydration is associated with an increased risk of acute kidney injury and the worse outcome of neurologic diseases, acute stroke, and in-hospital mortality in acute decompensated heart failure patients [7,8,9,10]. The UK DRIE study found that 48% of older people living in long-term care were dehydrated [2]. Another meta-analysis of 44 studies found that the prevalence of low-intake dehydration in older adults was 22.6% [11]. Other negative effects on human health caused by dehydration include an increased metabolic rate, inflammation, and degenerative changes, and it is associated with the development of age-related chronic degenerative diseases [12]. Growing evidence suggests that dehydration may contribute to elevated serum creatinine, often in association with non-nephrotic protein [13].

There are many objective markers of dehydration, among which the direct measurement of serum osmolality is the gold standard. In the absence of readily available, directly measured serum osmolality, calculated osmolality is also effective [5]. Elevated serum osmolality has been shown to be an independent risk factor for the development of chronic kidney disease (CKD) [14]. The pathways between dehydration and renal function decline have gradually been revealed [15]; however, few studies have investigated whether high serum osmolality causes renal function decline and CKD development in relatively healthy people. Therefore, the aim of this study was to investigate the prevalence of dehydration and the relationship between serum osmolality and renal function decline and CKD development or progression in the general population.

## 2. Materials and Methods

### 2.1. Study Design and Study Subjects

This study was a large-scale, single-center, retrospective cohort study to clarify the relationship between chronic dehydration and the risk of renal function decline. Using the database at the Chang Gung Memorial Hospital, Yunlin, Taiwan, we analyzed the medical records of subjects who underwent annual regular health check-up from 2016 to 2019. A total of 4449 subjects were included. These standardized annual healthy check-ups were provided to the general populations, and all blood samples were examined in the same laboratory. All of the subjects who attended the check-ups were healthy, and they provided a general history of any comorbidities. The study design allowed us to identify the relationship between calculated serum osmolality and both renal function decline and CKD development or progression in apparently healthy people. This study was approved by the Ethics Committee of our hospital (Institutional Review Board number: 202201041B0).

### 2.2. Definitions

Renal function decline was defined as a decrease in the estimated glomerular filtration rate (eGFR) from 2016 to 2019. The definition of CKD or CKD progression was based on the initial eGFR of the subject. For those with an eGFR ≥ 60 mL/min/1.73 m^2^ at presentation, CKD was defined as having an eGFR < 60 mL/min/1.73 m^2^ recorded between 2016 and 2019. For those with an eGFR < 60 mL/min/1.73 m^2^ at presentation, CKD progression was defined a decrease in their eGFR by 50% or <15 mL/min/1.73 m^2^ between 2016 and 2019. Proteinuria was measured in a spot urine sample using a urine dipstick test. We defined 1+, 2+, and 3+ as abnormal proteinuria in this study. A history of diabetes was self-reported by subjects before examination.

Serum creatinine was measured using colorimetric methods. Serum osmolality was calculated as follows: 2 × (Na + K) + BUN/2.8 + Glucose/18. The eGFR was calculated using the modified MDRD formula as follows: 175 × (creatinine)^(−1.154) × (age)^(−0.203) × [1.21 if black] × [0.742 if female].

### 2.3. Statistical Analysis

Continuous variables were expressed as the mean ± standard deviation (SD), depending on their distribution. For comparisons involving multiple groups, quantitative variables were subjected to an analysis of variance (ANOVA) followed by post hoc tests. Categorical variables were presented as counts and percentages, and were compared using the chi-square test or Fisher’s exact test. Statistical significance was defined as *p* < 0.05. To identify risk factors for CKD or CKD progression, both simple and multiple logistic regression analyses were conducted. Variables with *p* < 0.05 in the univariate analysis were included in the multiple logistic regression model. Cumulative event curves were estimated using the Kaplan–Meier method and compared using log-rank tests. To qualitatively assess the nonlinear relationship between a decline in renal function and serum osmolality levels, adjusted for age and sex, restricted cubic spline (RCS) analyses with log transformation were performed.

All statistical analyses were conducted using SPSS version 20 for Windows (IBM, Armonk, NY, USA) and R version 4.2.3 (R Foundation for Statistical Computing, Vienna, Austria).

## 3. Results

### 3.1. Data Collection

**Figure 1:**
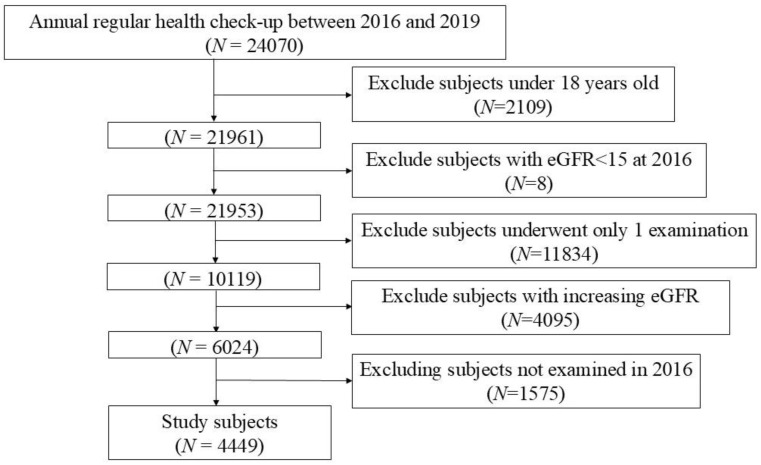
Flow diagram of enrollment.

We conducted a retrospective analysis of the medical records of 24,070 individuals who participated in annual medical examinations between 2016 and 2019 (Figure 1). Of these individuals, we excluded those who were <18 years of age as of 2016 (*N* = 2109), those with preexisting CKD stage V (eGFR < 15 mL/m/1.73 m^2^) (*N* = 8), and those who underwent only one examination between 2016 and 2019 (*N* = 11,834).

Furthermore, 4095 subjects who had an increase in their eGFR during the follow-up period and 1575 subjects who did not undergo an examination in 2016 were subsequently excluded. This resulted in a final cohort of 4449 subjects who were included in the study to elucidate the potential relationship between chronic dehydration and a decline in renal function over a 4-year period.

### 3.2. Characteristics of the Study Subjects

The study cohort was categorized into quartiles based on their osmolality levels in 2016 (Table 1). There were 1112, 1116, 1112, and 1109 individuals in the first-, second-, third-, and fourth-quartile groups, respectively, with average osmolality values of 293.26 ± 2.65, 297.45 ± 0.86, 300.40 ± 0.90, and 304.95 ± 2.70 mOsm/KgH_2_O. Table 1 presents the baseline demographic characteristics of the enrolled subjects. In all quartiles except the first, where a higher proportion of female subjects was observed, the distribution of sex was balanced. Age, body mass index (BMI), systolic blood pressure (SBP), and blood urea nitrogen (BUN) significantly increased with higher osmolality levels. The ages in the first-, second-, third-, and fourth-quartile groups were 40.12 ± 13.86, 44.28 ± 15.48, 50.69 ± 17.48, and 61.22 ± 15.47, respectively. The average BMIs in the four groups were 23.84 ± 4.19, 24.34 ± 4.16, 25.01 ± 4.01, and 25.84 ± 4.08 kg/m^2^, respectively, the average SBPs were 116.86 ± 17.22, 121.71 ± 18.14, 126.11 ± 18.00, and 132.96 ± 18.78 mmHg, and the mean BUN levels were 10.57 ± 3.21, 12.05 ± 3.38, 13.54 ± 3.68, and 16.96 ± 5.97 mg/dL. Conversely, the eGFR significantly decreased with increasing osmolality, with the eGFR values in the four groups being 96.79 ± 19.01, 91.26 ± 16.18, 87.44 ± 17.92, and 79.04 ± 18.56 mL/min/1.73 m^2^, respectively. In contrast to the other groups, the fourth-quartile group had elevated levels of ALT, total cholesterol, and proteinuria, and a history of diabetes mellitus. In addition, 63 (5.7%) subjects in the fourth-quartile group had a history of diabetes and 182 (16.5%) subjects had proteinuria. Smoking, serum sodium, and the triglyceride level showed no significant association with osmolality.

A total of 194 subjects (17.5%) in the 4th osmolality group reached the primary endpoint of CKD or CKD progression, a statistically significant difference compared to the 39 (3.5%), 36 (3.2%), and 90 (8.1%) subjects in the first-, second-, and third-quartile groups, respectively.

### 3.3. Univariate and Multivariate Analyses

To identify the independent factors associated with CKD or CKD progression, a total of 17 baseline characteristics (Table 1) were analyzed. In the univariate analysis, the variables with prognostic value are shown in Table 2. In the multivariate analysis, we excluded BUN due to its collinearity with osmolality. The result showed that independent risk factors for CKD or CKD progression were age (odds ratio (OR): 1.078, 95% confidence interval (CI): 1.067–1.090, *p* < 0.001), SBP (OR: 1.012, 95% CI: 1.004–1.020, *p* = 0.002), osmolality (OR: 1.034, 95% CI: 1.004–1.066, *p* = 0.025), uric acid (OR: 1.318, 95% CI: 1.204–1.442, *p* < 0.001), proteinuria (OR: 2.090, 95% CI: 1.521–2.871, *p* < 0.001), and a history of diabetes (OR: 2.662, 95% CI: 1.620–4.376, *p* < 0.001). On the other hand, high-density lipoprotein cholesterol was a protective factor with an OR of 0.985 (95% CI: 0.973–0.997, *p* = 0.013) (Table 2 and Table 3).

### 3.4. Cumulative CKD Incidence or CKD Progression

**Figure 2:**
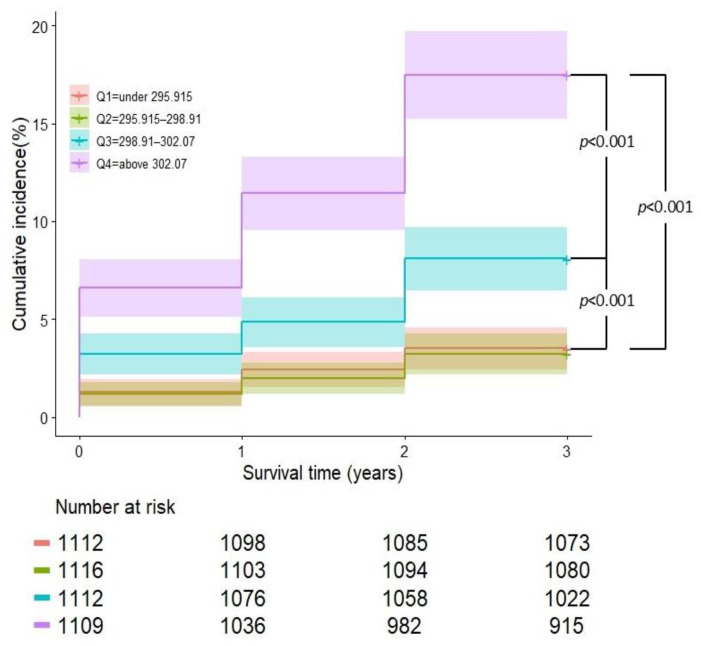
Cumulative incidence rate of CKD or CKD progression, quartile by osmolality.

The cumulative incidence rates of CKD or CKD progression in the four osmolality quartile groups were 3.5%, 3.2%, 8.1%, and 17.5%, respectively (Figure 2). There were no statistical differences between the first- and second-quartile groups (*p* = 0.710). However, the figure clearly illustrates a notable increase in the risk within the third-quartile group (*p* < 0.001), which became even more pronounced in the fourth-quartile group (*p* < 0.001).

### 3.5. Restricted Cubic Spline Analysis

**Figure 3:**
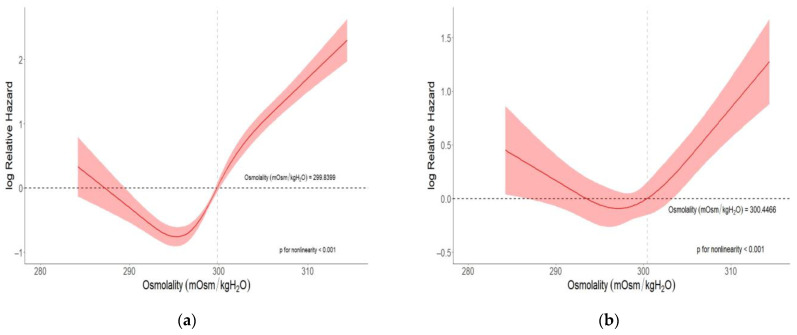
Estimated cubic spline transformation of the association between osmolality and CKD or CKD progression. (**a**) Univariate analysis. (**b**) Multivariate analysis after correction for age, sex, and diabetes history.

In the univariate restricted cubic spline analysis, the hazard ratio of CKD or CKD progression was markedly elevated when the osmolality level was above 299.8399 mOsm/KgH_2_O (Figure 3a). In the multivariate restricted cubic spline model, after adjusting for age, sex, and diabetes history, the risk of CKD or CKD progression was significantly elevated when the osmolality level was above 300.4466 mOsm/KgH_2_O (Figure 3b).

### 3.6. Renal Function Decline

**Figure 4:**
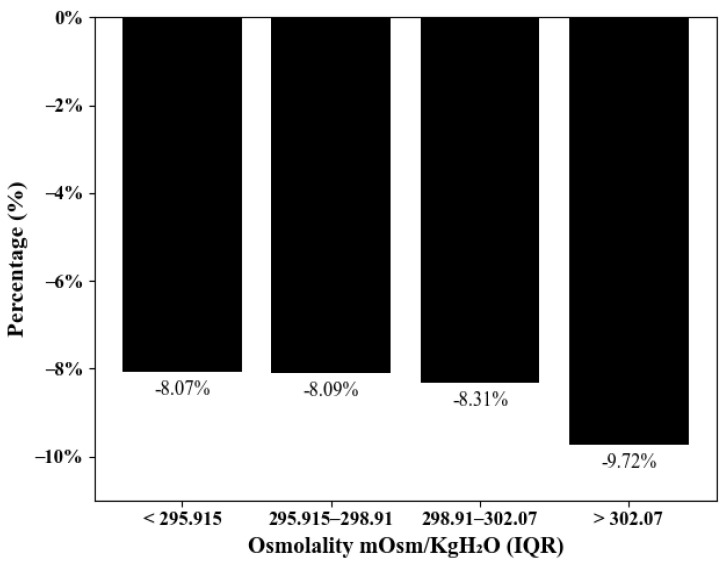
Renal function decline in 4-year period, quartile by osmolality.

A total of 2651 subjects underwent examinations in both 2016 and 2019, and their data were used to compute the average decline in renal function over the 4-year period (Figure 4). The subjects were classified into four groups based on their osmolality levels in 2016. After 4 years of follow-up, the third- and fourth-quartile groups had declines in their eGFR of 8.31% and 9.72%, respectively, while the first- and second-quartile groups had declines of 8.07% and 8.09%. A significantly greater renal function decline was observed in the higher osmolality groups compared to the lower osmolality groups.

## 4. Discussion

### 4.1. Osmolality and CKD or CKD Progression

Our results demonstrated that serum osmolality could predict the decline in renal function and development of CKD. The increased risk was independent of multiple other well-known risk factors, including age, SBP, hyperuricemia, proteinuria, and a history of diabetes mellitus. However, it is important to note that dehydration is not solely associated with high osmolality values. Dehydration can be classified into isotonic, hypertonic, and hypotonic types based on fluid loss and sodium concentrations. This classification can provide a clearer understanding of the relationship between dehydration and renal function [5].

Recent studies have consistently shown that chronic or recurrent dehydration is associated with chronic activation of the renin–angiotensin system, endothelin, vasopressin, and aldose-reductase–fructokinase pathway, which can subsequently lead to obesity, metabolic syndrome, hypertension, and CKD [15]. A Japanese study also reported an association between high osmolality and CKD [14]. In the present study, we found a significant difference in annual renal function decline with increasing serum osmolality, and also observed a significant increase in the risk of CKD when serum osmolality exceeded 300 mOsm/KgH_2_O.

### 4.2. Risk Factors for CKD or CKD Progression

Obesity is a traditional risk factor for CKD [16,17]. In the Framingham Offspring study cohort [18], a higher BMI was associated with a higher risk of developing CKD in a mean follow-up period of 18.5 years. The mean BMI was 26.3 kg/m^2^ in the non-CKD group and 27.4 kg/m^2^ in the CKD group. The Hypertension Detection and Follow-Up Program [19] also reported a significantly higher incidence of CKD in overweight and obese groups. The mean BMI was 22.7 kg/m^2^ in the ideal group, 27.4 kg/m^2^ in the overweight group, and 34.7 kg/m^2^ in the obese group. However, another study found no significant relationship between BMI and the incidence of CKD, in which the mean BMI was 23.88 kg/m^2^ [20]. In our study, BMI was not found to be an independent risk factor for CKD in multivariate analysis. The highest BMI (25.84 ± 4.08 kg/m^2^) was in the fourth-quartile osmolality group. The impact of obesity on the incidence of CKD may be more pronounced in populations with a higher BMI.

Different from previous studies [14,21], serum sodium was not the decisive content of osmolality in our analysis. According to reports from the Ministries of Health of Japan and Taiwan, Japanese people consume more salt than Taiwanese people. The daily sodium intake among the Japanese population declined from 5182 mg in 1995 to 3828 mg in 2019 [22]. However, the daily sodium intake among Taiwanese adult males was 2558–4140 mg and 2194–3196 mg in females [23]. Lower dietary sodium intake may consequently have a diminished impact on renal function decline.

BUN is another well-known risk factor for CKD [14,20,24]. In our study, BUN showed a trend of an increasing association with osmolality and CKD in the univariate analysis. Among the factors used to calculate serum osmolality, BUN was the only factor significantly associated with the risk of CKD.

Impaired fasting glucose has been associated with renal hyperfiltration in the general population [25]. However, it was not causally associated with CKD development in a study of a non-diabetic Mendelian population [26]. In our study, we observed a trend of an increase in fasting glucose levels in the high osmolality group. However, fasting glucose was not independently associated with the risk of CKD in the multivariate analysis.

An association between smoking and the progression of CKD was reported in a prospective Korean CKD cohort [26]. In addition, the risk of CKD progression was higher in the subjects who smoked more than 15 pack-years. Moreover, in the general population, smoking was also related to incident CKD in a dose-dependent manner [27]. In our study, smoking history was not an independent risk factor for CKD or related to serum osmolality. However, we lacked the data on the current or ever smoking status and the smoking load.

### 4.3. Future Prospectives

Some devices can assess the body’s fluid status by measuring salivary conductivity, which is highly compatible with serum osmolality [28]. These rapid, real-time, and easy-to-administer screening tools can be used to help improve serum osmolality. Future, prospective studies could investigate whether diet and lifestyle interventions can improve osmolality and whether they have an impact on renal function.

### 4.4. Limitations

This study has several limitations. First, this is a retrospective study with inherent limitations such as potential data inaccuracy, challenges in establishing causation, selection bias, and a lack of experimental controls. Second, we did not have access to HbA1c levels. Even though we recorded serum glucose, this is not sufficient to diagnose diabetes mellitus or to represent the long-term condition of diabetes mellitus control. Third, we provided recommendations after the health examinations, potentially leading to improved results at the follow-up visit. Fourth, as we lack data on dietary habits, the impact of subjects’ fluid, salt, and protein intake on osmolality and CKD or CKD progression remains unclear. Finally, our study lacks records of the subjects’ fluid status, which limits our ability to perform subgroup analyses based on this factor.

## 5. Conclusions

In conclusion, this study provides valuable insights into the associations among serum osmolality, dehydration, and renal outcomes in a general population. The findings underscore the importance of addressing dehydration as a modifiable risk factor for renal function decline and CKD development. Future prospective studies could explore interventions and further elucidate causal relationships.

## Figures and Tables

**Table 1:** Demographic data and clinical characteristics, quartile by osmolality.

	Q1	Q2	Q3	Q4	*p*-Value
Number of subjects	1112	1116	1112	1109	
Sex (male/female)	353/759	547/569	590/522	524/585	<0.001
Age	40.12 ± 13.86	44.28 ± 15.48	50.69 ± 17.48	61.22 ± 15.47	<0.001
Body mass index (kg/m^2^)	23.84 ±4.19	24.34 ± 4.16	25.01 ± 4.01	25.84 ± 4.08	<0.001
Smoking (%)	9/1112 (0.8)	14/1116 (1.3)	12/1112 (1.1)	9/1109 (0.8)	0.655
SBP (mmHg)	116.86 ± 17.22	121.71 ± 18.14	126.11 ± 18.00	132.96 ± 18.78	<0.001
ALT (U/L)	20.91 ± 20.28	23.99 ± 24.38	25.13 ± 20.26	26.38 ± 25.34	<0.001
Osmolality (mOsm/kgH_2_O)	293.26 ± 2.65	297.45 ± 0.86	300.40 ± 0.90	304.95 ± 2.70	<0.001
BUN (mg/dL)	10.57 ±3.21	12.05 ±3.38	13.54 ±3.68	16.96 ±5.97	<0.001
eGFR (mL/m/1.73 m^2^)	96.79 ± 19.01	91.26 ± 16.18	87.44 ± 17.92	79.04 ± 18.56	<0.001
Sodium (mEq/L)	140.26 ± 2.04	140.22 ± 1.98	140.34 ± 1.96	140.19 ± 2.24	0.543
Potassium (mEq/L)	4.09 ± 0.28	4.18 ± 0.29	4.19 ± 0.29	4.23 ± 0.35	<0.001
Total cholesterol (mg/dL)	181.61 ± 34.97	184.96 ± 34.52	184.16 ± 35.19	187.35 ± 36.41	0.002
HDL (mg/dL)	54.82 ± 13.21	53.03 ± 12.45	51.49 ± 12.42	51.73 ± 12.47	<0.001
LDL (mg/dL)	107.37 ± 30.90	111.22 ± 30.51	110.75 ± 29.76	111.66 ± 31.43	0.004
Triglyceride (mg/dL)	108.94 ± 147.82	110.96 ± 94.12	111.71 ± 74.33	114.77 ± 86.26	0.618
Fasting glucose (mg/dL)	91.05 ± 16.47	92.67 ± 14.80	97.59 ± 22.37	105.00 ± 30.43	<0.001
Uric acid (mg/dL)	4.93 ± 1.39	5.42 ± 1.49	5.63 ± 1.49	5.69 ± 1.43	<0.001
Proteinuria (%)	97/1112 (9.0)	98/1116 (8.9)	111/1112 (10.1)	182/1109 (16.5)	<0.001
History of diabetes (%)	16/1112 (1.4)	12/1116 (1.1)	19/1112 (1.7)	63/1109 (5.7)	<0.001
Primary endpoint (%)	39/1112 (3.5)	36/1116 (3.2)	90/1112 (8.1)	194/1109 (17.5)	<0.001

SBP = systolic blood pressure, BUN = blood urea nitrogen, ALT = alanine aminotransferase, eGFR = estimated glomerular filtration rate, HDL = high-density-lipoprotein, LDL = low-density lipoprotein.

**Table 2:** Risk factors for CKD or CKD progression, univariate analysis.

Parameter	Odds Ratio (95%CI)	*p*-Value
Sex	0.869 (0.701–1.079)	0.204
Age	1.092 (1.082–1.102)	<0.001
Body mass index (kg/m^2^)	1.103 (1.077–1.129)	<0.001
SBP (mmHg)	1.042 (1.035–1.048)	<0.001
ALT (U/L)	1.005 (1.001–1.008)	0.007
Osmolality (mOsm/kgH_2_O)	1.178 (1.150–1.207)	<0.001
BUN (mg/dL)	1.252 (1.223–1.282)	<0.001
eGFR (mL/m/1.73 m^2^)	0.861 (0.850–0.872)	<0.001
Sodium (mEq/L)	0.986 (0.921–1.055)	0.680
Potassium (mEq/L)	1.451 (1.022–2.058)	0.037
Total cholesterol (mg/dL)	0.998 (0.995–1.001)	0.184
HDL (mg/dL)	0.957 (0.947–0.966)	<0.001
LDL (mg/dL)	0.995 (0.991–0.998)	0.004
Fasting glucose (mg/dL)	1.016 (1.013–1.019)	<0.001
Uric acid (mg/dL)	1.475 (1.360–1.561)	<0.001
Proteinuria	3.640 (2.822–4.695)	<0.001
History of diabetes	5.525 (3.631–8.408)	<0.001

SBP = systolic blood pressure, BUN = blood urea nitrogen, ALT = alanine aminotransferase, eGFR = estimated glomerular filtration rate, HDL = high-density lipoprotein, LDL = low-density lipoprotein.

**Table 3:** Risk factors for CKD or CKD progression, multivariate analysis.

Parameter	Odds Ratio (95%CI)	*p*-Value
Age	1.078 (1.067–1.090)	<0.001
SBP (mmHg)	1.012 (1.004–1.020)	0.002
Osmolality (mOsm/kgH_2_O)	1.034 (1.004–1.066)	0.025
HDL (mg/dL)	0.985 (0.973–0.997)	0.013
Uric acid (mg/dL)	1.318 (1.204–1.442)	<0.001
Proteinuria	2.090 (1.521–2.871)	<0.001
History of diabetes	2.662 (1.620–4.376)	<0.001

HDL= high-density-lipoprotein.

## Data Availability

Data supporting the findings of this study are available from the corresponding author, Peng, on request.
